# An exploration of the Indonesian lay mental health workers’ (cadres) experiences in performing their roles in community mental health services: a qualitative study

**DOI:** 10.1186/s13033-024-00622-0

**Published:** 2024-01-17

**Authors:** Herni Susanti, Helen Brooks, Ice Yulia, Heni D. Windarwati, Estin Yuliastuti, Hasniah Hasniah, Budi A. Keliat

**Affiliations:** 1 Mental Health Nursing Department, Faculty of Nursing Universitas Indonesia, Depok, Indonesia; 2 Mental Health Research Group, Division of Nursing, Midwifery and Social Work, School of Health Sciences, Faculty of Biology, Medicine, and Health, University of Manchester, Manchester Academic Health Science Centre, Manchester, UK; 3 Mental Health Nursing Department, Faculty of Health Sciences, Universitas Brawijaya, Malang, Indonesia; 4 Institute of Technological Sciences in Health PKU Muhammadiyah Surakarta, Surakarta, Indonesia; 5 Politeknik Kesehatan Kemenkes Aceh, Aceh Besar, Indonesia

**Keywords:** Lay health worker, Cadre, Roles, Community mental health service, Qualitative research

## Abstract

**Background:**

Volunteers trained to support community mental health programs in Indonesia are known as ‘mental health cadres.’ These are lay people trained to provide basic support for people with mental illness in their local communities. The role of cadres in community mental health services is to provide health promotion activities and support for people with mental illness, such as home visits and family assistance. Their contribution can potentially address the challenges health services currently face in remote and resource-limited settings. However, little is currently known about implementing this form of the lay workforce and the experiences of mental health cadres in Indonesia in particular. This study aimed to explore the experience of cadres when performing their roles in community mental health services in Indonesia from the cadres’ perspective.

**Methods:**

The study employed a descriptive qualitative design. Purposive sampling was employed to recruit cadres with at least one year of experience handling those diagnosed with schizophrenia across four geographical areas in Java and Sumatra, Indonesia. Data were collected utilising focus groups undertaken between July and November 2020. Due to COVID-19 restrictions, eight focus group sessions for mental health cadres were carried out virtually via Zoom and non-virtual, facilitated by local moderators. Data were analysed using thematic analysis.

**Results:**

The study involved 71 cadres in four regions: Aceh, Jakarta, West Java and East Java. The majority of participants were looking after their families with a minimum of high school-level qualifications. Four themes were interpreted from the data: (1) Motivation for volunteering, (2) The role of cadres in supporting mental health services, (3) Training and support needs in carrying out cadre roles, and (4) Barriers and facilitators to the implementation of cadre roles in local communities.

**Conclusions:**

Cadres reported a motivation to help people improve their mental health and reduce the stigma associated with mental illness. Cadres also contributed to secondary and primary prevention of mental illness with some limitations. This study’s results are relevant to those wishing to understand and optimise the implementation of lay workforces in resource-limited settings.

**Supplementary Information:**

The online version contains supplementary material available at 10.1186/s13033-024-00622-0.

## Introduction

Mental problems are a global public health concern. In 2017, at least one in ten people experienced a mental illness or an estimated 792 million (10.7%) people live with the condition across the world [1]. In particular, as many as 20 million people have had schizophrenia (severe mental disorder) [2]. The impact of mental illness is significant and far-reaching. Those diagnosed with mental illness often experience poor quality of life, which is characterised by feelings of distress, lack of autonomy control, low self-esteem, hopelessness, and diminished activity, they also have productivity loss and poverty in all indicators (lower income, lower education, food insecurity and unemployment) [3–6]. The consequences of mental illness are not only limited to patients but also have a profound impact on the community through economic costs and lost human capital potential [7, 8]. In addition, there is a high number of cases of socially excluded people with mental illness. This is due to the environmental, institutional, and attitudinal barriers faced by people with mental illness, especially those associated with social inclusion and stigmatisation [9–11].

The National health survey by the Indonesian government showed that 6.7 per 1000 families have a family member diagnosed with schizophrenia, of which around 14% experience confinement [12]. Around 0.31% of people live in Indonesia with schizophrenia, placing Indonesia above the mean prevalence of schizophrenia in the WHO Southeast Asia region [2]. Aceh is a region in Indonesia with the prevalence of family members diagnosed with schizophrenia exceeding the national average of approximately 8.7 per 1000 families. Schizophrenia is the leading cause (96.12%) of inpatient patient visits at referral (tertiary) hospitals in Aceh Province [13]. In contrast, East Java, DKI Jakarta, and West Java are provinces that have people living with schizophrenia below the national average of 6.6, 6.4, and 5.5 per 1000 families, respectively; however, 0.11% of the DKI Jakarta population living with schizophrenia, only 41% of cases have been recorded and handled [12, 14]. Meanwhile, in East Java and West Java, although the prevalence is lower than the national average, caregiver problems, relapse, restraint, and stigma in people living with schizophrenia are still a concern [15–18].

The World Health Organization emphasises that mental illness has caused one in four people to live with a disability [19]. Mental illness was also included in the top ten disability-adjusted life years in people aged 15 to 29 years in the last decade in Indonesia [20]. Indonesia has conferred upper-middle income status [21], but at 2.8% of the GDP, Indonesia’s total health expenditure is one of the lowest in Southeast Asia [22]. Further, only 2% of health expenditure is directed towards mental health, and 66% of mental health expenditure is allocated to tertiary care [23]. Moreover, about 48.9% of people with schizophrenia seek treatment at healthcare facilities and take routine medication in the last month. Staff shortages and imbalances in the professional skills mix have hindered the development of community mental health services and resulted in an over-reliance on hospital care [24, 25]. Indonesia is a large archipelago with high levels of geographical and socio-economic diversity, which is considered to have exacerbated this treatment gap through inequitable access to appropriate and effective care [26].

The World Health Organisation has endorsed task-shifting as the optimal way health systems can reduce treatment gaps to improve community health [27]. This has been explained as a process in which specific tasks are moved (where appropriate) from specialist health workers to professionals with a different or lower level of training or to lay workers provided with training and support to deliver care without the need for formal health education [27]. Research has demonstrated that such approaches can improve the efficiency of services, improve satisfaction with care, and enhance cultural sensitivity and uptake of interventions [28–30]. The evidence base for task-shifting is broad, with demonstrable benefits evidence across several studies [31, 32]. Mental health delivery, however, is highly context-specific and in-depth explorations of such approaches to support people living with mental illness in Indonesia are lacking.

One example of a task-shifting approach within Indonesian health services is community members’ involvement in the mental health system (known as ‘cadres’ in Indonesia). Cadres are lay workers who contribute to health promotion activities and support formal health workers in providing services in their local communities, for example, home visits, facilitating patient referrals in the community, and assisting the family [33, 34]. Mental health cadres are recruited through socialisation on cadre formation by local health workers and community leaders. Mental health cadres are volunteers who work flexible and regular hours (one to two times per month) [35–37]. However, cadres receive government incentives as accommodation subsidies, which are received every 3–6 months [38, 39]. In collaboration with local community health centres, the Regional Public Health Office and Provincial Health Office provide these cadres with centralised training and supervision programmes about guidance community mental health programs. Community health centres in Indonesia called *Pusat Kesehatan Masyarakat* (Puskesmas) are primary healthcare facilities that provide individual and public health services in their geographical area, with a particular focus on promotive and preventive programs to improve the community’s health status [40]. Concerning mental health, cadres are expected to support the delivery of mental health programs designed by community health centres. Mental health cadres are the front line of community-based services providing psychoeducation, supporting the early detection of mental problems, and monitoring medication adherence with the help of families through a programme of regular home visits [33, 37].

The commitment to strengthening mental health services at a national level is vital in Indonesia, supported by government policy Number 8, 2019 [41], by the Indonesian Minister of Health Regulation concerning community empowerment in the health sector, and lay workers are likely to be well-placed to understand the needs of people living in their local areas [42]. Given their acceptance by and position within local communities, they are also optimally positioned to challenge negative societal attitudes towards those with mental problems, which are ubiquitous in Indonesia and the increasing demand for mental health treatment [43, 44].

Broader evidence suggests that people in Southeast Asia may be more receptive to preventive and treatment services provided by lay workers [45, 46]. In Indonesia specifically, qualitative research and randomised controlled trials demonstrate that task-shifting may be a potentially useful way to strengthen mental health systems [34]. In addition, the perceived advantages of cadres’ involvement in mental health services from the point of view of families, people with schizophrenia, and the surrounding community in Indonesia have also been highlighted in recent qualitative research [44]. Syntheses of evidence from other countries, however, draw attention to potential barriers that might impact the utility of lay workers, including patient acceptability, financial sustainability, health professional confidence, and managerial infrastructure [47, 48]. It is, therefore, imperative to develop in-depth contextual information about the experience of delivering such roles to drive forward the implementation of this workforce in Indonesia.

Despite the potential value and increasing use of these roles within mental health services, the experience of delivering such activities from the cadres’ perspective remains poorly understood. As a result, the improvement of public mental health through cadres’ involvement is hampered by the absence of in-depth data regarding cadres’ perceptions of their roles, barriers and facilitators to role delivery, and training and support needs. Therefore, this study aims to understand the mental health cadres’ experience when they perform their role of handling people with schizophrenia in the community mental health services from their own perspectives.

## Materials and methods

This study employed a descriptive qualitative approach [49]. This study was part of the more extensive research project, Exploration of the Role of Mental Health Workers (Cadres) in Community Mental Health Services in Indonesia, which explored the experiences of families and people with schizophrenia who received support from mental health cadres. This study focused on the experience and role of cadres in community mental health services in Indonesia from the cadres’ perspective.

### Participants and recruitment

The mental health cadres in this study were recruited from four provinces: Jakarta, West Java, Aceh, and East Java. This study was conducted in Jakarta, West Java, Aceh, and East Java because they are pilot projects for implementing the Community Mental Health Nursing Program in Indonesia and having well-established programs with trained health workers. Also, Aceh has a high prevalence of household members with mental health problems compared to other provinces in Indonesia. In comparison, the other two areas have a prevalence of mental health problems below the national average. Inclusion and exclusion criteria for participants were: (a) participants are at least 18 years old, (b) able to communicate well in Indonesian or regional languages (Acehnese/Javanese/Sundanese) which participants and researchers understand, and (c) participants were eligible to take part in the study if they had received centralised training and had at least one year of experience as a cadre handle for those diagnosed with schizophrenia.

Information relating to potential participants was obtained from the database of included Puskesmas. First, the researchers determined whether the potential participants were eligible based on inclusion/exclusion criteria, and once this was ascertained, potential participants were contacted via phone or WhatsApp to assess their interest in taking part in the study. If they were interested in participating, participants were sent an information sheet and consent forms and another appointment was made to discuss participation in more depth before making a final decision about participation. All potential participants contacted were willing to participate, and none declined.

In total, 71 mental health cadres took part in eight focus groups. Each group ranged from 6 to 12 participants. The average age of the participants was 42 years old, and the participants had, on average, 54 months of experience as a cadre. Participants reported spending an average of 2.82 h per week undertaking their role. Most participants were females (*n* = 66), married (*n* = 66), and were obliged to look after their families. The majority held high school qualifications.

### Data collection

A series of focus groups were conducted to obtain in-depth data based on participants’ experiences of being a mental health cadre, focusing on their experience when performing their current roles, day-to-day activities, training and support needs and barriers and facilitators to undertaking their roles. Focus groups were selected due to their ability to capture an exchange of viewpoints and promote interactive discussion of phenomena of interest [50].

Data collection was carried out in the midst of the Covid-19 pandemic, i.e., from July to November 2020. As a result, the data collection reached saturation in the eight focus group discussions (two groups in Aceh, two in Jakarta and West Java, and four in East Java). Details of the distribution of these groups are presented in Table 1. Two delivery methods were therefore adopted: offline (non-virtual) and online (virtual) focus group discussions. Due to local restrictions, participants in Aceh and East Java were permitted to engage in face-to-face discussions in study sites, facilitated by local moderators with the adoption of strict health protocols such as physical distancing, wearing face masks, and making ventilation available to allow air circulation. In contrast, in Jakarta and West Java, in-person focus groups were not permitted, therefore, focus groups were carried out remotely using Zoom, a video conference platform. The participants of the remote focus groups also followed local health protocols by attending the session in their own houses and not in public spaces.

On average, each group’s discussion lasted from 60 to 90 min. Focus groups were undertaken in Bahasa Indonesia, the most common language used in the Jakarta and West Java. In the other two provinces, the participants discussed the research topic in their own language (Acehnese and Javanese). Each discussion was facilitated by local moderators who had experience conducting focus groups or qualitative research and were familiar with or had access to the research settings where cadres were present. The local moderators facilitating the focus group were professional mental health professionals and senior lecturers with qualitative experiences. Before the study, they participated in meetings about focused group interview guidance, short training, and briefings on perception and skills in conducting focus groups to minimise inconsistencies in data collection. The researcher records on an audio recorder for non-virtual focus group discussion and a Zoom recorder for virtual focus group discussion and writes field notes immediately after the focus group discussion sessions.

Focus group interview guidelines were developed through a process of studying relevant literature regarding the role of lay workers in mental health services [35, 51–53] and through consultation with the wider study team (Appendix 1). Facilitators were briefed on the focus group protocol in two series of online meetings before undertaking focus groups. We set additional facilitators to attend each focus group session to provide technical assistance and to implement the distress policy. If a stressful condition is experienced by one of the participants in the FGD, the facilitator will give time for the participant to express their feelings in the group. Then, the participant will also be asked whether they want to stop the FGD or continue. If they wish to continue, the facilitator will focus on the FGD guidelines again. If they want to stop, they will be asked to stay in the room to cool off and if they want to talk individually after the FGD is over. Since the facilitator is a psychiatric nursing specialist and an academician, the participant will be given specialist nursing actions to calm them down. During all focus group discussion sessions, all participants did not feel stressed, so the facilitators did not implement the distress policy.

The participants filled out a demographic form before the commencement of the focus groups, including their age, gender, educational background, marital status, length of experience, and hours spent working as a cadre per week.

**Table 1:** The distribution of focus group participants across provinces

Focus group name	Research sites	Number of participants	Gender of participants	The average age of the participants	The average length of experience	Method of data collection
FG1	East Java	10	Female: 8 Male: 2	41 years old	28 months	Non-virtual
FG2	East Java	10	Female: 10 Male: -	46 years old	28 months	Virtual (zoom)
FG 3	East Java	10	Female: 8 Male: 2	47 years old	69 months	Virtual (zoom)
FG 4	East Java	9	Female: 9 Male: -	44 years old	100 months	Non-virtual
FG5	Aceh	10	Female: 10 Male: -	32 years old	50 months	Non-virtual
FG 6	Aceh	12	Female: 12 Male: -	31 years old	46 months	Non-virtual
FG 7	West Java	9	Female: 7 Male: 2	44 years old	93 months	Virtual (zoom)
FG 8	Jakarta	6	Female: 5 Male: 1	50 years old	68 months	Virtual (zoom)

### Data analysis

Thematic analysis is carried out to inductive analyse the data [54]. The thematic analysis involves six phases of coding and theme development. Transcripts were first read a number of times by analysts to ensure immersion in the data. Next, the first and second authors independently coded all transcripts before meeting to agree on a final set of codes. During this meeting, codes were organised in an iterative process involving removing duplicate codes and unifying similar ones into higher-order codes. Codes were then organised into potential themes which were considered appropriate to answer the research question. The initial coding framework developed during this stage was shared with other research team members for further refinement before the agreement was reached amongst the team that the identified themes fully reflected the data from all eight focus groups. The final stage of the analysis was writing the manuscript, which involved providing thick descriptions of the included participants, writing in-depth accounts of interpreted themes, and selecting quotes from the raw data to illustrate interpretations. Member checking was done by consulting research participants to clarify any ambiguous data from the transcripts [55]. This was done once during the analysis process to allow clarification in local languages about any ambiguities within the transcripts.

The trustworthiness of qualitative data analysis is based on its credibility, transferability, dependability, and conformability. The credibility of the focus groups was achieved through peer debriefing by working together with all researchers, including a non-Indonesian investigator who was not immersed in local culture and practices. This investigator examined and provided feedback on the transcripts, reports, and a general methodology focused on applying thematic analysis.

All researchers reviewed and agreed on the themes which emerged from the study. Transferability refers to the extent to which others can apply qualitative research findings to their own contexts [56, 57]. The transferability of qualitative research, including the current focus groups, can be accomplished through different strategies, such as providing ample descriptions regarding the research context, processes, participants, and participant-researchers’ relationships [57, 58]. These accounts assist the reader in deciding whether the findings can be transferred to other populations or settings.

The dependability of the analysis was enhanced through regular meetings with all researchers, and this was helpful in revealing the researchers’ biases, assumptions, and data misinterpretation. Conformability addresses the neutrality of the naturalistic inquiry [57, 59]. This lies in the perspective that the interpretation, as far as possible, is derived from the data, representing the situation being researched rather than the investigator’s beliefs, imagination, or biases. The conformability of the focus group was enhanced by using data from different contexts, which helped confirm the findings [60].

## Results

Four main themes were interpreted from the data: motivation for volunteering, the role of cadres in supporting mental health services, training and support needs in carrying out cadre roles, and barriers and facilitators to the implementation of cadre roles in local communities.

### Motivation for volunteering – the salience of altruism and giving back to communities

The dominant motivation for wanting to become a cadre reported by participants was the desire to help people with mental illness and to provide support to local communities generally. It appeared that this motivation could be increased by seeing first-hand the impact of mental illness and mental health-related stigma in their communities. The details and complete supporting quotes can be seen in Appendix 2.

*“Over time, my conscious has awakened. Every time I see these ill people being ostracised, then I say to myself … I must be enthusiastic, I must not despair, I must be enthusiastic about helping them so that they can interact with their neighbours and can be independent people.” (FG 3, P7, Female, 45 years old, 120 months of experience, East Java)*.

Participants emphasised this desire to help people with mental illness as reflecting the wider values, cultures, and traditions inherent to Indonesia. These values are central to the founding principles of the country, which can be summarised into a term called *gotong royong* or mutual cooperation, caring for others, and sharing burdens.

*“Our intention as mental health cadres is to help fellow human beings become better. The development of loved ones (people with mental disorders) becomes our strength as mental health cadres” (FG1, P6, Female, 44 years old, 36 months of experience, East Java)*.

Others reported feeling motivated to support those with mental illness because there was limited other support available for them.

*“In my opinion, there is no support for this patient. But because we are used to doing social work, so seeing the patient’s condition motivated us to want to help. We feel pity for his situation. If possible, how we can help to make him recover … Like today. Thank God, my patient has been able to help his mother by selling rice cake. He often goes to the market and does his shopping on his own.” (FG 4, P3, Female, 50 years old, 98 of experience, East Java).*

This desire to help others was not only limited to helping people with mental illness but also to supporting the cadres’ own families, neighbours, acquaintances, and particularly those at risk of mental health difficulties. The cadres acquired the skill to support those who might be vulnerable to mental health difficulties from a short training given by mental health professionals from Puskesmas and supplemented by scholars or academicians from related fields. The training included a session to practice their communication skills with mental health patients and their families.

*“I can help friends with a mental illness. We can encourage them. We will help them so they can be handled by the mental health service. The main thing is that we have a lot of experience as mental health cadres. It is not only for motivating the patients to get up. So, from our conscious and our hearts, all of us feel the call to help our friends” (FG2, P1, Female, 45 years old, 36 months of experience, East Java)*.

The participants conceptualise their role as cadres not only as a job and responsibility but also as a calling. This was particularly true for female cadres who often described this role as one of a nurturing maternal can be seen from the majority of cadres being female and how they see this role as nurturing and mothering.

*“Here in Bongkot, there are many people whose children have problems, we from the cadres, from the beginning, are willing to help these children to be independent, it’s like that. So, they can be responsible, so there’s no burden, we feel responsible.” (FG3, P4, Female, 35 years old, 60 months of experience, East Java)*.

Additional reported motivations for becoming cadres included a desire to have responsibility within communities and acknowledgement from the government for their contributions, such as rewards in the form of a certain amount of money or access to free medical treatment. However, not all participants have been receiving rewards in the form of money from the government for their contributions.

*“Sometimes we feel like we hope to get a reward, maybe in some kind like a small amount of money” (FG2, P7, Female, 41 years old, 36 months of experience, East Java)*.

### The role of cadres in supporting mental health services – cadres as an extension of formal health services

Participants described the range of activities they undertook as part of their role as mental health cadres. Almost all participants in this study reported that their most regular activities were undertaking home visits either independently or together with health workers such as nurses, as these visits are instigated by *Puskesmas* or initiated in response to requests from the community. As such requests came unpredictably, it means that there were no precise working hours for them, and they were expected to be always ready.

*“We have activities every week and every month. We visit a patient when we get a report from the neighbours that he or she relapses. When there is a new patient, we usually go to this patient immediately. As a cadre, we must be ready to visit patients at any time, we don’t work only for certain days or certain months… we have to be ready” (FG 2, P4, 48 years old, 18 months of experience, East Java)*.

Several participants reported undertaking mental health detection activities in their local communities and described their role as an extension of formal primary health care services in this regard. Cadres’ perceived role was to provide data for health services about those who had mental illness or who were at risk of developing mental illness.

*“One of the roles of cadres is to carry out early detection. We group those who are still healthy, those who are at risk, or those who have mental problems. We are an extension of the Puskesmas (community health centres) officers to the village so that it is easier for the officers to get data on people who have problems or who are at risk”. (FG 5, P2, Female, 23 years old, 24 months of experience, Aceh)*

Another reported activity of cadres was mobilising community members to support public health education. Several participants stated that they encouraged people with mental illness, their families, and broader community members to participate in public health education activities, such as regular mental health counselling organised by Puskesmas. This is normally conducted in common spaces available such as Posyandu (Integrated Health Centre) and mosques, where the families and community members, in general, were invited to come.

*“We invite and encourage the people in the neighbourhood to understand about mental problems in the village. We also invite them to come if there is counselling at Meunasah [mosque] or Posyandu, and these are not only dedicated for mental people but also for pregnant women and the elderly who need information”. (FG 5, P3, 34 years old, 24 months of experience, Aceh)*

Supporting people with mental illness and families to seek help from formal health providers was also identified by a number of participants as central to the role of mental health cadres. Cadres could also refer patients directly to health services or liaise with village officials to ensure people could access treatment. In terms of potential roles, there is something about contributing to sustaining recovery from mental illness for people.

*“Coordination is usually with the village officials, if a patient is really to be taken to health services, then we call the health worker for coordination to be referred, usually we refer the patient to a hospital or Puskesmas” (FG 3, P2, female, 45 years old, 62 months of experience, East Java)*.

Before the COVID pandemic, the participants carried out various activities targeting mental health patients and their families, such as regular home visits. However, during the global COVID-19 pandemic, there was a reported shift in the role and associated activities of the cadres due to COVID-related restrictions and the fear of infection felt by all cadres. However, due to the pandemic, participants reported complaints from families about changes in activities and could not meet directly with health cadres. As a result, the cadres conveyed the importance of face-to-face activities and direct communication to help people with mental disorders to be better.

*“They (people with mental disorders) can not just take medicine, and it must be balanced with direct meeting activities so that they can communicate and be happy; if they are happy, the body’s immunity goes up. But because of Covid-19, there have been many changes, especially many families who have complained. Why are activities changing like this?” (FG1, P7, Female, 34 years old, 24 months of experience, East Java)*.

Moreover, this shifting was also felt by the families since the cadres no longer came for home visits.

*“There are many things changed during this pandemic. We are doing fewer activities now. And many families complained about this. They wondered why it became like this.” (FG3, P7, 45 years old, 120 months of experience, East Java)*.

### Training and support needs in carrying out cadre roles

Participants coalesced in their desire for more structured training and supervision programmes in formal health services to increase their roles’ range and scope. Participants also felt it was essential to have regular meetings between cadres within and outside their areas to share learning and promote reflection on their roles.

*“We need cadre meetings like this [focus group], and then we are given materials of knowledge… as we don’t get a training for several days, well, at least we have a training like this cadre meeting.” (FG1, P2, Female, 45 years old, 36 months of experience, East Java)*.

Participants also described a need for greater coordination between cadres and within villages to increase community understanding of their roles.

*“In essence, this is what we have to do with each other… cooperation between us as cadres, cooperation with superiors from the Puskesmas, as well as cooperation from patients, especially their families. So, yes, there must be support from above [Puskesmas], then the community should also understand our work as cadres.” (FGD 9, P5, Male, 58 years old, 24 months of experience, Jakarta)*.

Other expectations are related to financial compensation for the cadres themselves who supported the treatment of people with mental illness, which was often seen as financially and emotionally burdensome. Participants also described a need for financial resources to support people with mental illness and their families to support participation in meaningful activities within local communities. They mentioned the potential of people with mental illness to be trained in several crafting skills like sewing, carpentry, and crafting batik.

*“One of our patients has a carpentry skill like making a shoe rack and cupboard. We need financial support to make them productive and train them so that they can sell their products” (FG1, P6, Female, 44 years old, 36 months of experience, East Java)*.

Some participants also considered formal legal recognition of the role of cadres to be important in terms of developing the role in the future. This would increase the legitimacy of their roles within communities but also might help to leverage the requisite financial resources described previously.

*“The cadres themselves need to be considered, in this case, the legality of the government.” (FGD 2, P7, Female, 41 years old, 36 months of experience, East Java)*.

Participants felt they needed more regular monitoring and supervision from trained health professionals in villages and an increase in the number of professionals who were able to provide this mentoring and support. It was also expected that health workers should accompany cadres when visiting people with mental illness and their families in their neighbourhoods more frequently so that family members felt more cared for by primary health care centres and so that access to medication was facilitated.

*“In my opinion, what is needed is the medical personnel, ma’am, for example, when they can monitor more often, you know, it can be from the Puskesmas. They can monitor more often. So, as cadres, when we are monitored frequently, this makes me feel like I am being cared for.” (FG 7, P2, 67 years old, 18 months old of experience, West Java)*.

### Barriers and facilitators to the implementation of cadre roles in local communities

Participants in this study identified barriers and facilitators to implementing their roles in local communities. In terms of barriers, several participants described obstacles that they attributed as deriving from people with mental illness, including lack of insight into illness, difficult behaviours, family or people with mental illness refusing support from cadres, and societal stigma of being cadres.

*“In my opinion, there are pluses and minuses in being cadres. The good thing is we know more about health, it keeps us close to the community, continue to get to know other people too… such as health workers at the Puskesmas, in hospitals, or anywhere else we are free to do something. But the bad side is to be a cadre, it’s like being ridiculed.” (FG7, P7, Female, 45 years old, 60 months of experience, West Java*).

In terms of stigma, it is also worth noting that some of the cadres and cadres’ families refer to people with mental illness as “lazy” and “crazy”. Cadre’s families also ask about why cadres take care of people with mental disorders, which reflects potentially stigmatising views about those with mental illness.

*“Apart from responsibilities, we can do good deeds for later in the hereafter. For the family, it’s normal, maybe my husband seems a bit objectionable because he wonders why I have to take care of crazy people.” (FG7, P7, Female, 45 years old, 60 months of experience, West Java)*.

The majority of participants also reported difficulties in engaging with families of people with mental illness, including home visit refusal, reluctance to speak openly about mental illness, and not confiding in cadres because of a fear of negative judgements within the wider community. The family refuses support from the cadres because the family feels ashamed of the cadres because the family has members who experience mental illness, and the family is afraid of being stigmatized by society.

*“A general challenge is that family with a mentally ill member. They are embarrassed, ma’am. They are ashamed to see us as cadres” (FG8, P3, Female, 51 years old, 54 months of experience, Jakarta)*.

There were also a number of barriers highlighted by the cadres themselves. These included a lack of the requisite skills and knowledge about mental health to provide care effectively within communities and limited financial resources to support their activities. Often participants described having to use their own resources to facilitate access to care for people with mental illness which were quickly depleted. In addition, there were specific reported fears amongst cadres taking part in focus groups about how to engage with patients experiencing positive symptoms of schizophrenia, such as hallucinations and delusions and those displaying aggressive or difficult behaviours.

*“I think all cadres’ knowledge is limited” (FG3, P8, Female, 40 years old, 60 months of experience, East Java)*.

The lack of trained health professionals to support their roles further exacerbated the identified barriers.

*“In that subdistrict, there is one person (a nurse) who works in high demand… the number [of nurses] is lacking… the number of visits is too high, while some villages have not finished yet. Once, I called the nurse. It was at 9 PM at night. Yet, it was really difficult to contact him, until finally, I waited for an hour…” (FG4, P3, Female, 50 years old, 98 months of experience, East Java)*.

Participants identified the demand for mental health cadre services according to the number of patients. However, the lack of experience directly dealing with people with mental disorders was one of the obstacles expressed by the cadres in carrying out their roles, and this was due to the absence of people with mental illnesses in the cadres’ area. On the other hand, several participants expressed their hope of increasing the number of mental health cadres.

*“So I don’t have any hands-on experience. It’s only theory. So far, there are no people with mental disorders in my area because I live in housing. So far, in terms of coverage, it has been lacking. So for me, personally, I have never handled it.” (FG 7, P5, Male, 51 years old, 159 months old, West Java)*.

*“We need more support from other parties and, if possible, add cadres, increase the number of cadres so that everything can be handled” ((FG7, P3, female, 39 years old, 24 months of experience, West Java)*.

Participants also identified a range of facilitators to undertake their roles as cadres. These included support from their own family members, which had the potential to mitigate against the strain associated with the identified barriers. Support from fellow cadres and the community’s proximity was also considered important in terms of successfully implementing their roles. This included both informational and emotional support relating to the role as cadres.

*“The support also comes from our cadre friends; they want to help with information or something else. This is helping me. We make a cooperation.” (FG8, P4, Female, 37 years old, 96 months of experience, Jakarta)*.

Where support was available from trained health professionals, particularly trained nurses, this was also viewed positively and as instrumental to successful undertaking of their daily activities. A small number of cadres also reported receiving support from village officials in terms of providing incentives and transportation/subsistence costs, which are considered a major facilitator to implement their roles.

*“The main support is from Nurse X. Without her, we all cannot be in our beloved team, the truth is that her assisting role is very noble” (FG3, P7, Female, 45 years old, 120 months old, East Java)*.

## Discussion

The finding of the exploration of the perspective of mental health cadres was related to the cadre’s experiences and roles when committing to participating in community mental health services in Indonesian culture. The key finding motivating cadres was the importance of altruism and giving back to the community and the culture and traditions inherent in Indonesia. Altruism is an attitude of giving help without expecting anything in return, and the motive of wanting to dedicate themselves to others without expecting anything in return has been found in the volunteer community in Indonesia [61]. Indonesia has a lot of cultures, one of which is the tradition of helping each other, and a high level of altruistic behaviour shows this. The high level of altruism in Indonesia potentially encourages people to become lay mental health workers and play a role in community health services for people with mental disorders.

Cadres are an extension of formal mental health services. In carrying out their roles, cadres have routine tasks such as home visits, early detection of mental problems, supporting health education, and supporting patients and families, which were delayed due to COVID-19. COVID-19 has consequences for cadres in carrying out their roles, including outreach, auxiliary care, and prevention of mental problems in the community and for people with mental illness, which decreased the utilisation of access to mental health services [62, 63]. This can be overcome by training and developing the skills of health cadres [63], which was in line with the key findings in this research about the importance of monitoring and supervising mental health professionals and the importance of training mental health cadres to optimise their roles.

In addition, acknowledgement from the government and financial compensation need to be improved to build on their roles. Research conducted by Singh et al. (2015) showed that the use of inadequate incentives could cause a loss of intrinsic motivation in community health workers, the acknowledgement deemed valuable by community health workers in the form of gifts and appreciation [64]. Thus, it is essential to maximise community appreciation and altruistic value in optimising the role of mental health cadres. Receiving support in terms of incentives and transportation/subsistence costs was considered a significant facilitator to implementing their mental health cadres carrying out their roles. In community-based mental health services, the infrastructure and non-financial resources are often inadequate and are usually minimally funded [65].

The role of stigma in dealing with mental problems is well known, which is an obstacle for cadres in carrying out their role. The stigmatisation of people with mental illness causes barriers to access to good quality health services [66]. The potential emersion of stigma from either the cadres themselves or the wider community is one of the barriers to implementing the cadres’ role in this study. Frequently associating people with mental disorders with violence, instability, and danger, as well as a lack of knowledge about mental health in society, reinforces the stigmatisation of mental illness [67]. Stigmatisation is moderated by knowledge, knowledge and perceptions of mental illness related to stigmatisation [58, 59]. Understanding stigma can reduce its impact on mental health [68], so it is essential for mental health cadres to receive training to increase their knowledge and skills.

In conducting their roles, there is a broad coverage of cadres’ activities from this study that shows their valuable role in increasing access to mental health services through contributions from the community itself. The World Health Organization (WHO) defined civic engagement as a development process where people in the community are made possible and motivated to collaborate on health-related issues and enhance well-being for the betterment of the community [69]. In Indonesia, cadres’ participation in improving community health has legal standing and is explained in the Indonesian Minister of Health Regulation concerning community empowerment in the health sector. It is stated that cadres perform as community mobilisers, public health educators, documentation and report if there are health problems [41]. As stated in the study in Indonesia, civic engagement is likely to serve as a way to tackle health-related challenges by developing person-centred care models [70]. In this study, the concept of civic engagement, along with the benefits identified, such as lower use of funds and human resources, has been proven to be implemented quite well by these cadres. This is due to the fact that when compared to the high-income countries, whose financial resources and health access to mental health services were sufficient [71]. In Indonesia, the perceived care of people with mental illness was not merely related to health centres and health professionals but also associated with the broader community. Moreover, people in rural settings viewed the scheme of lay health workers or cadres positively [44].

It was disclosed that the cadres’ roles were beneficial as an extension of the health workers. This can be supported by the findings of research from the study in rural society in Indonesia, which stated that their roles were helpful because they have better skills for dealing with schizophrenia people [72]. This expression needs to be translated by positioning health workers as partners in improving mental health in the community. As stated by one participant, the cadres wanted the health workers to be their companions- working hand-in-hand in carrying out their duties. At least they hope there is more frequent monitoring so they do not feel left out or ‘abandoned’. In terms of support, the results of this study indicated that the assistance and direction of mental health personnel, especially nurses, are essential. This gives advantages to mental health services because they have reliable assistance in achieving program targets. A study in the United States found that the service given by lay health workers was significantly valued by the patient and considered a factor in improving health outcomes [73]. In contrast, in this study, cadres experienced difficulties engaging with families of people with mental illness due to a fear of negative judgments in the broader community. This is related to the stigma in society, which causes patients and families to be reluctant to seek treatment [11].

This research provides an in-depth understanding of cadres’ experience of undertaking their roles in Indonesian health services, which is currently lacking despite this role having been established for the last two decades. The study gains its strength from the in-depth approach to data collection and sample, which was distributed across geographical regions in Java (Western and Eastern Java) and Aceh in Sumatera, Indonesia.

Meanwhile, the weakness of this research was that data collection carried out during the pandemic reduced access to cadres in the Jakarta and Bogor-West Java areas, so the number of cadres involved was much less than in other areas. Furthermore, due to the pandemic situation, focus groups in these two areas were conducted online, and this, although not obvious in the data collected, is likely to have affected the dynamics between participants during the discussions and the resultant data [74]. Still, in the end, the ideas for these two methods were a high degree of overlap. Moreover, the geographical variation between the research locations and the backgrounds of lay health workers in urban and rural areas might cause the results of this study not to apply there. Although lay health workers from rural and urban areas obtain training from the Regional Health Office and receive incentives such as accommodation assistance [38, 39, 75], they differ in regional accessibility. Rural areas have broad and remote areas with more challenging access than urban ones [76].

## Conclusions

Cadres reported a strong motivation to help people improve their mental health and reduce the stigma associated with mental illness at a community level in Indonesia. Data on the activities of cadres showed that they also supported the families of people with mental illness and made significant contributions to providing psychoeducation at a community level. In addition, cadres reported also contributing to secondary and primary prevention of mental illness with some limitations. To optimise the role of cadres within communities, a more structured programme of training and support from trained health professionals is required, along with the provision of financial resources in the form of individual remuneration, transportation costs and resources to support meaningful activities for people with mental illness.

### Electronic supplementary material

Below is the link to the electronic supplementary material.

Appendix 1 - Focus group guideline, Appendix 2. Participants quoted

## Data Availability

The data that support the findings of this study are available on request from the corresponding author [Herni Susanti]. The data are not publicly available because they contain information that could compromise the privacy of research participants.
