# Supplementary material for: An exploration of the Indonesian lay mental health workers’ (cadres) experiences in performing their roles in community mental health services: a qualitative study
Source: Int J Ment Health Syst. 2024 Jan 17;18:3. doi: 10.1186/s13033-024-00622-0 (PMC10792806; doi:10.1186/s13033-024-00622-0)
Supplement: Supplementary file 1 — Appendix 1 - Focus group guideline, Appendix 2. Participants quoted [file 13033_2024_622_MOESM1_ESM.docx]

## Supplementary Materials

Appendix 1 - Focus group guideline

1. **Opening questions**
2. What are the roles of mental health cadre that you have run so far?
3. What activities do you do?
4. How long does it take to carry out these tasks?
5. **Core question**
6. What do you think about your role as a mental health cadre so far?
7. What are the things that support and hinder you in carrying out your role as a mental health cadre so far?
8. What are your expectations about your role as a mental health cadre in the future?
9. What do you need to make these expectations come true?

Appendix 2. Participants quoted

| No | Quotes | Category | Theme |
| --- | --- | --- | --- |
| 1 | *"We become mental health cadres as a form of our service to the community, especially for mentally ill patients who need special attention, so they are able to get quality services, not be ostracised in society, there is no rejection in the society or no discrimination". (FG2, P6, Female, 50 years old, 12 months of experience, East Java)* | Provide support to local communities | **Motivation for volunteering – the salience of altruism and giving back to communities** |
| 2 | *"Something that moved me to become a cadre is a sense of caring. You know, in our village, even when we cook, many people will know it [the smell] then we will share… especially for these people… so we care about each other." (FG 3, P1, Male, 57 years old, 68 months of experience, East Java)* | Reflecting the wider values, cultures and traditions inherent to Indonesia |  |
| 3 | *“... our children, our beloved children… they did everything, rampaging, swearing, (laugh) it’s like that Ma’am, now, alhamdulillah he already works...”* *(FG3, P1, Male, 57 years old, 68 months of experience)* | Nurturing and maternal roles |  |
| 4 | *“We have been given the trust of the village, we have been appointed to be mental health cadres, so we must be responsible. As you have said that you are ready, then you have to take responsibility.” (FG 4, P1, Female, 38 years old, 98 months of experience, East Java)* | Responsibility within communities |  |
| 5 | “*In addition to giving health insurance or free medical treatment for patients who need it, maybe the government can also give it to us cadres” (FG2, P3, Female, 40 years old, 48 months of experience, East Java)* | Acknowledgement from government |  |
| 6 | *"As a cadre, I'm trying to advise patients to get treatment regularly. The role of the cadre is also to help explain to the family regarding the treatment, so we will guide them” (FG 8, P4, Female, 52 years old, 21 months of experience, Jakarta)* | Supporting people with mental health problems and families | **The role of cadres in supporting mental health services** |
| 7 | **“***This may be for the next, us as cadres, our responsibility will be further increased in visiting patients who are already better”(FG7, P3, Female, 39 years old, 24 months of experience, West Java)* |  |  |
| 8 | *“Before covid, we always performed home visits. But now, during the pandemic, we communicate with patients using a phone. We always video call them. We fear doing a face-to-face visit, and we always follow health protocols.” (FG9, P3, 51 years old, 54 months of experience, Jakarta)* | Shifting roles performance during the pandemic and Fear of COVID-19 infection |  |
| 9 | *“During the pandemic, we cannot freely carry out our activities. Because we are susceptible to covid and we fear getting infected too.” (FG8, P7, 45 years old, 60 months of experience, West Java)* |  |  |
| 10 | *"We don't want the training once a year, ma'am. We want every six months." (FG6, P3, 25 years old, 46 months of experience, Aceh)* | Structured training and supervision | **Training and support needs in carrying out cadre roles** |
| 11 | *"So, for the recovery period, patients should be given profitable activities so they become more productive in their community. In the beginning, we need support so that such moneymaking activities can be created, and then they are able to participate… even though it is small ...." (FGD 4, P2, 46 years old, 98 months of experience, East Java)* | Need for financial support |  |
| 12 | *“For example, in Bandungrejo, a patient is very skilled in sewing, and she has started to receive orders like sewing clothes. It will also help the family’s financial condition” (FG2, P1, Female, 45 years old, 36 months of experience, East Java)* | Potential people with mental health problems crafting skills |  |
| 13 | *"Maybe health workers can visit at least every three months or every six months to patients and their families… for asking about the patient’s condition, accompanied by cadres, of course, so that the family will feel being cared. Because these people usually feel ostracised, ma'am." (FG 7, P5, Male, 51 years old, 159 months old, West Java)* | Needs for monitoring and supervision from health professionals |  |
| 14 | *“We really need a companion because I am* the *only device to support these patients, but the health workers have to assist them physically. It's more important for the patients to get medicine at once while I'm mostly just working around it. So, that's all, I want a companion from the health team." (FG 4, P2, Female, 46 years old, 98 months old, East Java)* |  |  |
| 15 | *"Our difficulty when meeting X (a service user) is that he is a bit indifference… as if he doesn’t want to be visited, does he?”(FG9, P3, Female, 51 years old, 54 months of experience, Jakarta)* | Rejecting meeting cadres | **Barriers and facilitators to the implementation of cadre roles in local communities** |
| 16 | *"But now, ma'am, the problem seems to be that he doesn’t want to take medicine. He believes that he is not ill. When he was asked why he was not taking the drugs? He said that he is tired of taking medicine all time” (FG 7, P2, Female, 67 years old, 18 months of experience, West Java)* | Lack of insight into illness |  |
| 17 | *“The problem is… not all mentally ill people can communicate well. Sometimes they immediately go wild and even carry a weapon, which makes us afraid (FG 4, P2, Female, 46 years old, 98 months of experience, East Java)* | Difficult behaviour |  |
| 18 | *"I visited his family, they didn't accept me, ma'am, the family was silent. I was there together with a nurse. At that time, every household did not want to be visited. Yes, that's the challenge” (FG3, P1, Male, 57 years old, 68 months of experience, East Java)* | difficulties in engaging with the families |  |
| 19 | *"Then the second is that sometimes families who are not cooperative mean that they are told to take medicine, the family doesn't pay attention to it, even if the medicine is taken, it doesn't drink." (FG 3, P6, Female, 51 years old, 60 months of experience, East Java)* | reluctance to speak and not confiding in cadres |  |
| 20 | *“It's quite far to get medicine to the Puskesmas [with a service user] , so inevitably… use your own money… drive your own motorbike. So, we have to pay for them by ourselves to get the medicine, then go back and forth to the Puskesmas for the sake of the patients” (FG7, P6, 48 years old, 360 months of experience, West Java )* | Limited financial resources |  |
| 21 | *“My husband doesn't reject me, ma'am. I mean, it's normal. She said that I am ok, I am a cadre, then I can go [when I am needed] (FG1, P2, Female, 54 years old, 36 months of experience, East Java)* | Support from the cadres family |  |
| 22 | *“I happen to walk around the area every day. So, even though I keep going around, asking questions, I thank God, that I have many sisters even though I don’t give anything to them, I just give support and the spirit from the cadres… the important thing is that I am healthy, asking for prayers from other mothers. (FG7, P1, Female, 26 years old, 36 months of experience, West Java)* |  |  |
| 23 | *“Our head of village has budgeted for our meals in any mental health activity that is carried out in public places” (FG 2, P3, Female, 40 years old, 48 months of experience, East Java)* | support from village officials |  |
